# ASSOCIATION BETWEEN SCAPULAR DYSKINESIA AND SHOULDER PAIN IN YOUNG ADULTS

**DOI:** 10.1590/1413-785220162405142225

**Published:** 2016

**Authors:** HUGO MACHADO SANCHEZ, ELIANE GOUVEIA DE MORAIS SANCHEZ, LARISSA INGREDDY TAVARES

**Affiliations:** 1. Universidade de Rio Verde (UniRV), Rio Verde, GO, Brazil.

**Keywords:** Shoulder pain, Shoulder, Scapula, Dyskinesias

## Abstract

**Objective:**

To analyze the position of the scapula and its influence on shoulder pain.

**Methods:**

In this study, 30 sedentary young adults of both genders, aged 20-35 years were evaluated. The sample was divided into two groups with the same number of subjects, one group with shoulder pain and the other pain free. The analysis of the positioning of the scapula in six angles of shoulder abduction was taken 0º, 30º, 60º, 90º, 120º and 180º.

**Results:**

Comparison the left and right scapular movements in males of the pain group, there was a significant difference at 30º (p = 0.018) and 120º (p = 0.04). Comparing the right and left shoulders in the pain group, there was a significant difference at 0º (p = 0.03).

**Conclusion:**

This study concludes that changing the positioning of the scapula affects shoulder pain in sedentary young adult males at certain specific positions. Level of Evidence III, Study of non consecutive patients; without consistently applied reference ''gold'' standard.

## INTRODUCTION

Shoulder diseases have aroused much interest among medical professionals. This is mainly because the shoulder is responsible for implementing most of the movement and positioning of the upper limb.^1^

The shoulder is considered fairly stable due to its joint anatomy, especially in the glenohumeral joint, which has great mobility and low stability, making it necessary for the synchronic and constant harmony between all the structures that maintain the normal biomechanics. Any change that compromises their structure and function makes that complex joint a target of numerous conditions.^2^

The shoulder complex has been reported in the literature as a compound of joints. The scapulothoracic joint is one of the most important joints of this complex, being classified as a functional joint, since it allows the scapula to slide along the chest and participate in all the shoulder's complex movements.^2^ The movements performed by the scapula are: abduction, adduction, upward rotation, downward rotation, elevation, depression, protraction (with elevation of the lower angle of the scapula) and retraction.^3^ The scapula must move in a coordinated manner with the humerus, keeping the humeral head rotation axis and synergy movement, which is called scapulohumeral pace.^4^

The glenohumeral joint is the most mobile and least stable of all joints of the human body.^5^ In the shoulder girdle, the glenohumeral joint, also called scapulohumeral joint, is considered the main joint of the shoulder complex and, when this joint complex works harmonically, it allows upper limbs large range of motion, making the shoulder the most mobile joint of the human body.^6^

The movements of the glenohumeral and scapulothoracic joint should be in tune in order to provide a perfect harmony during the execution of lifting, abduction and flexion movements of the shoulder, providing balance to muscle activation pattern and a wide range of motion. Any changes in the scapulothoracic pace leads to the so called scapular dyskinesia.^7^

The purpose of the scapular movement is to achieve a movement relation capable to maintain the glenoid cavity in a good position that enables it to receive the humerus head, increasing, thus, the range of motion and ensuring that the concomitant movement of the scapula allows the muscles to act on the humerus, in order to maintain a satisfactory relationship to achieve full and harmonic motion.^5^

Scapular dyskinesia is considered any change occurring in the scapulothoracic pace, which causes a change in the position, scapular movements or normal mobility of the scapula relative to the thorax.^8^ It is the term used to describe visible changes in the scapular position in movement patterns and these changes in the scapular movement patterns have been associated to some shoulder injuries.^9^ Dyskinesia does not exactly designate where the dysfunction occurs, since there are several factors that may lead to changes in the scapular position, such as poor posture, excessive resting posture, thoracic and cervical kyphosis, lordosis, clavicle fractures, injuries to the acromioclavicular joint, instabilities, arthrosis and changes in the function of the muscles that control the scapula.^7^

It is believed that the scapula plays a major role in shoulder function and any change in its positioning directly influences muscle strength and stability of the shoulder girdle. Changes in shoulder girdle mobility have been related to shoulder pain, a condition characterized by symptoms in the joints, muscles, tendons and bursae, all of them involved with shoulder motion. The onset of shoulder pain is variable and may occur without any direct cause or may be related to a trauma or repetitive movements, and pain may often cause limitation to activities.^4^

In view of the exposed, this study aimed to analyze the position of the scapula and its effect on non-traumatic shoulder pain onset.

## MATERIALS AND METHODS

This is a descriptive observational cross-sectional study, where we used a non-probabilistic convenience sample consisting of young sedentary adults of both genders, aged 20-35 years old. The sample consisted of 30 individuals, who were divided into two groups each consisting of 15 individuals, two groups of volunteers, one presenting shoulder pain and the other asymptomatic.

This study was approved by the Ethics Research Committee of University of Rio Verde, under protocol number 096/2012. A Free and Informed Consent form was prepared, in which the research objectives were exposed to all participants, guaranteeing anonymity and confidentiality of the data, in accordance to the ethical aspects ensured by Resolution 196/96 involving human subjects of *Conselho Nacional de Ética em Pesquisa*.

To be part of the research, individuals of both genders were aged 20-35 years and not physical active. In one group, the volunteers had no shoulder pain and participants of other group had shoulder pain. In neither groups, however, volunteers had other associated injuries such as traumatic injuries, shoulder dislocations or previous clavicle or scapula fractures. In order to check for shoulder injuries, the Neer test and the arm wrestling test were performed.^10^ Those who were not able to perform the shoulder abduction movement, or showed any of the above described shoulder injuries were excluded from the sample.

Data were collected in a large, air-conditioned room. The researcher filled out an evaluation form at the assessment environment, containing individual personal data and questions related to physical exercise and movement that provoke any shoulder pain. The volunteers were instructed to stand at marked predetermined locations (adhesive tapes on the ground). Then, their bilateral upper and lower angle of the scapula were marked with stickers. The researchers were trained in palpation anatomy for the demarcation of the above mentioned points, in order to standardize the demarcation of anatomical accidents. 

**Figure 1:**
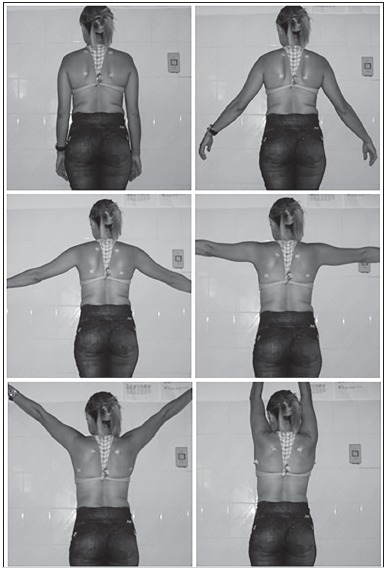
Positioning of scapula at the six shoulder abduction moments: 0°, 30°, 60°, 90°, 120°, and 180°.

Using a 10.1 megapixel digital camera, photos of the individual standing upright, with a nude torso facing the camera and making free active shoulders abduction movements were taken. Six images of the scapula were registered: 1^st^ photo, with pendant shoulder next to the body at 0°; 2^nd^ photo, with 30° shoulder abduction; 3^rd^ photo, with 60° shoulder abduction; 4^th^ photo, with 90° shoulder abduction; 5^th^ photo, with 120° shoulder abduction; and a 6^th^ photo, with maximum abduction without spine compensation (approximately 170-180°). (Figure 1) It is important to point out that at every change of position, the markers were repositioned bilaterally in the upper and lower angle of the scapula by the researchers who received proper training and had knowledge in palpatory anatomy.

In order to confirm the shoulder positions at the described amplitudes, a goniometer was used, and to locate the axis, the acromion was palpated, and approximately two fingers below it, the goniometer was positioned. The fixed bar was placed in the volunteer's torso facing the soil and the mobile bar accompanied the adduction movement, also following the back region of the arm.^11^

To capture all images (with no zoom), the camera was standardly placed at level and in line, being positioned with the aid of a tripod, at the hip of each volunteer and 2m away from them. During data collection performed by two researchers, one of them marked the scapula at each position change and the other took the photos. Remarking of anatomical points was essential, since surface adhesives were used, and skin movement did not follow the bone movements. Otherwise, images of points non-corresponding to bone accidents would have been recorded.

**Figure 2:**
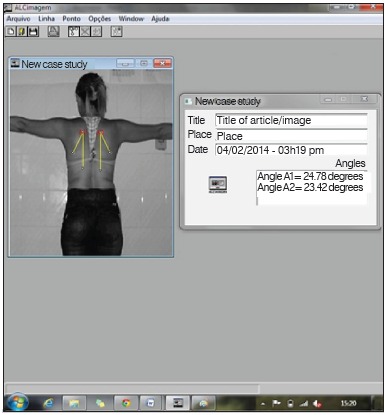
Calculation of scapular movement using the ACLimagem^(r)^ software.

After collecting the data, researchers transferred the photos to a computer for biophotogrammetry data analysis through the ALCimagem(r) software, where angular analyzes of the six positioning moments of the shoulder were performed, through visualization of the previously marked points. The angular analysis measured the lateral rotation of the scapula in the frontal plane in the six abduction positions of the arm. Two lines were drawn whose apex was the superior angle of the scapula. The first line was a longitudinal line in the direction of the spinal column and the second line joined the upper and lower angles of the scapula. (Figure 2) For greater reliability of the calculation of the scapular positioning, the procedure was repeated three times in each of the six positions and the average of three measurements was used for statistical analysis.

Statistical analysis was performed by applying the Shapiro-Wilks test, which found that the data were parametric. From this conclusion on, the Student *t*-test and the chi-square test were chosen with statistical significance postulated at 5% (p<0.05).

## RESULTS

We analyzed 30 individuals, divided into two groups, each containing 15 individuals. In the pain-free group, 11 were female and four male, with mean age 22.67 ± 4.93 years old; and in the pain group, nine were female and six male, with a mean age of 26.33 ± 5.20 years.

Table 1 describes the values ​​of the scapular movement in six different positions of all volunteers evaluated. Checking the result of descriptive statistics, the scapular movement was 6.95 ± 4.89° for the resting shoulder, 13.24 ± 8.86° for the shoulder at 30°, 23.24 ± 8.73° for shoulder abduction at 60°, 32.84 ± 8.24° at 90° abduction, 42.24 ± 9.30° at 120°, and 49.50 ± 7.56° for the maximum abduction, 180°.

**Table 1:** Mean values and standard deviation of scapular movements at the six positions analyzed (N=30).

Position	0°	30°	60°	90°	120°	180°
Mean	6.95	13.24	23.24	32.84	42.24	49.50
Standard Deviation	4.89	8.86	8.73	8.24	9.30	7.56

Comparing the scapular right and left movements in the male subjects of the pain group, a statistically significant difference was observed at 30° (p = 0.01) and 120° (p = 0.04), while in the other positions no significant differences were found. The same comparison made in female subjects found no statistically significant difference, and for the male subjects, the right scapula showed greater lateral rotation as compared to the left scapula at 30° and 120°. (Table 2)

**Table 2:** Comparison between scapular movements on the right and left sides of male and female individuals of the pain group at shoulder positions at 0^o^, 30^o^, 60^o^, 90^o^, 120^o^ and 180^o^ through the Student *t*-*test*.

Gender	Shoulder/ position	0^o^	30^o^	60^o^	90^o^	120^o^	180^o^
Male	Right	1.05±6.01	20.68±8.45	28.21±10.59	36.06±8.42	45.83±5.66	52.96±5.45
	Left	6.47±5.13	16.79±10 z\.22	27.68±11.58	38.18±8.10	49.19±4.72	57.17±5.87
	*p* value	0.10	0.01*	0.85	0.48	0.04*	0.20
Female	Right	8.66±6.39	13.45±11.93	24.06±9.25	33.40±10.24	42.33±13.67	49.30±11.16
	Left	5.94±5.21	14.32±10.69	23.17±9.08	30.18±9.15	40.97±7.32	50.32±4.80
	*p* value	0.17	0.81	0.74	0.53	0.65	0.79

* p < 0,05.

Comparing the right and left movements in the pain group, we found a statistically significant difference at 0° (p = 0.03), what lead us to conclude that the position of the right scapula is more lateralized as compared to the left scapula for the resting shoulder. It is inferred that the pain group showed postural change at 0°. In the other comparisons, no statistically significant differences were found, and the p values are as follows: 30°, p=0.65; 60°, p=0.69; 90°, p=0.79; 120°, p=0.78; and 180°, p=0.36. (Table 3)

**Table 3:** Mean values and standard deviation of shoulder position in the pain group at 0^o^, 30^o^, 60^o^, 90^o^, 120^o^, and 180^o^ and results of Student *t-test* (N=30).

Position	0^o^	30^o^	60^o^	90^o^	120^o^	180^o^
Right	9.61±5.93	16.34±10.6	25.72±9.33	34.47±9.67	43.73±10.64	50.76±8.92
Left	6.15±4.83	15.31±9.86	24.95±9.67	33.76±9.01	44.26±7.23	53.06±5.92
*p* value	0.03*	0.65	0.69	0.79	0.78	0.36

* p<0,05.

In the pain group 13 volunteers had pain in the right shoulder, and of these, 12 had right dominance, and one left-handed, while the two volunteers reporting pain in their left shoulder, one was left-handed and the other, right-handed. Applying the Chi-square test, we observed that the right shoulder showed statistically more pain than the left shoulder (p = 0.04). Moreover, it was also found that the pain side is at the dominant side in 14 out of 15 pain bearing volunteers, showing a statistical significance (p = 0.00), i.e., the dominant shoulder is statistically more painful.

In the comparison between the right and left movements in the group without pain, there was no statistically significant difference in any of the positions, i.e., the scapula moved in a likely manner in this group, and at 0°, p=0.96; 30°, p=0.52; 60°, p=0.72; 90°, p=0.82; 120°, p=0.83; and 180°, p=0.43. (Table 4)

**Table 4:** Mean values and standard deviation of shoulder position in the pain-free group at 0^o^, 30^o^, 60^o^, 90^o^, 120^o^, and 180^o^ and results of Student *t-test* (N=30).

Position	0^o^	30^o^	60^o^	90^o^	120^o^	180^o^
Right	5.99±3.66	11.21±6.27	21.58±7.11	31.89±5.67	40.25±9.25	46.35±6.79
Left	6.06±3.8	10.10±6.27	20.87±8.10	31.38±9.07	40.72±9.38	47.81±6.55
*p* value	0.96	0.52	0.72	0.82	0.83	0.43

In the comparative study between the values ​​found in pain and pain-free groups, we found no statistically significant difference in the comparisons between scapular position in each of the six angles and p = 0.08 in the comparison of the right scapula at 0°, p = 0.95 for the comparison of the left scapula at 0°, p = 0.12 for the comparison of the right scapula at 30°, p = 0.18 for the comparison of the left scapula at 30°, p = 0.19 for the comparison of the right scapula at 60°, p = 0.34 for the comparison of the left scapula at 60°, p = 0.41 for the comparison of the right scapula at 90°, p = 0.35 for the comparison of the left scapula at 90°, p = 0.26 for the comparison of the right scapula at 120°, p = 0.18 for the comparison of the left scapula at 120°, p = 0.13 for the comparison of the right scapula in 180°, and p = 0.07 for the comparison of the left scapula at 180°.

Through the six different angles analyzed in the pain group, nine of the volunteers reported greater pain at 120° angle totaling 60%, four reported pain at 90° totaling 26.67% and two volunteers reported greater pain at 180°, with a total of 13.33%.

## DISCUSSION

In the current study, we recruited individuals of both genders, which were separated into two groups. The mean age of the pain-free group was 2.67 ± 4.93 years, and the pain group was 26.33 ± 5.20 years. In a study by Santana et al.,^10^ in which swimmers were analyzed for the presence of scapular dyskinesia and/or shoulder pain, the average age of the group was 25.1 ± 4.7 years, ranging from 18 to 36 years. Pimentel et al.^12^ conducted a study among university handball players, which aimed to determine the prevalence of shoulder pain in these players, they analyzed male and female handball team players, who were non-federated student-athletes aged between 17 and 30 years. According to the information collected regarding the overall mean age of volunteers of all studies described, it is assumed that in the age group between 17 and 36 years, pain often does not relate to degenerative process of the glenohumeral and acromioclavicular joints.^13^

In this study we found that male subjects of the pain group had scapular changes in movements performed at 30° and 120° abduction. The scapular change at 30° may be due to the fact that during the contraction phase (0 - 30° abduction), the movement is greater in the glenohumeral joint, while the scapula searches a stable position, i.e. in the first 30° abduction or in the first 45° to 60° flexion, the scapula moves toward the spine or away from it, seeking a stable position in the thorax and, after reaching this stabilization, the scapula moves laterally, anteriorly and superiorly.^2^

From this information, we may suggest that the onset of scapular changes at 30° is related to the first moment of scapula movement in the so-called scapular-humeral pace, i.e., the first time when the scapulae is mobilized during abduction (from 30°), it loses the synergy in the scapular humeral pace and accelerates its lateral rotation, determining a scapular dyskinesia to this shoulder. Since, according to Silva,^8^ dyskinesia is any change occurring in the scapulothoracic pace that causes a change in position, scapular movement or normal mobility of the scapula in relation to the thorax. This statement is based on the fact that this change only occurs in painful shoulder of male volunteers.

According to Assunção and Vilela,^14^ the total abduction movement is 180° degrees and is the results of the abduction of the glenohumeral joint and the rotation of scapulothoracic joint, in which 120° of the movement comes from the glenohumeral joint and the other 60° from the scapulothoracic joint and other joints of the shoulder complex. When the abduction exceeds 110°, the movement starts to require other joints, such as the acromioclavicular and sternoclavicular joints. From this fact, it can be seen that the change of the scapular movement at 120° is due to a short deficit in the movement of the acromioclavicular joint and sternoclavicular, since in this amplitude, as reported, they should mobilize and when this does not occur and the range of motion is achieved, other joints are overloaded, in this case it was the scapulothoracic joint, since its mobilization was greater on the pain side of the analyzed volunteers.

In the study by Santana et al.,^10^ 36 swimmers were analyzed for the presence of scapular dyskinesia and/or shoulder pain. The mean time they practiced swimming was 10.3 ± 6.1 years. Most participants (47.2%) trained six times a week. Most individuals (86.1%) had a history of shoulder pain. Like the present study, the dominance showed predilection for the right shoulder, swimmers also had more pain in the right shoulder (58.1%). It was observed that the only left-handed participant who had shoulder pain, their pain was located in the dominant shoulder, and among right-handed participants, 76.6% had pain in the right shoulder.

This study also revealed that individuals with shoulder pain have a higher incidence of pain the dominant side; in the pain group, of 13 individuals with pain in the right shoulder, 12 had right dominance and one was left-handed, while the two participants reporting pain in the left shoulder, one was left-handed and the other right-handed. The right shoulder showed more pain than the left shoulder (p = 0.04); moreover, we also found that the pain side is the dominant side in 14 out of 15 individuals reporting pain, with statistical significance (p = 0.00), i.e., the dominant shoulder is statistically more painful. This high frequency of pain in the dominant shoulder suggests that excessive use predisposes to early fatigue by changing the pattern of muscle activation, culminating in a scapulothoracic dysfunction which is associated to pain.

In a study by Pimentel et al.^12^ among university handball players, which aimed to determine the prevalence of shoulder pain in these players, they analyzed male and female handball players at the Law School-USP. The athletes were non-federated student-athletes aged 17-30 years, height 1.50-2.00m and weight 45-100k. Regarding pain laterality, 11 players (64.7%) reported pain in the right shoulder; four (23.5%) on the left side and two (11.7%) in both shoulders, confirming data from a study with volleyball and handball players in which the dominant limb was predominantly the right and also the side most affected by glenoid labrum injury.

In the study of Ejnisman,^3^ 119 athletes with pain complaints in the shoulder area were evaluated, of which 95 (79.8%) were male, and 24 (20.2%) female. As for the athletes' category, 76 (63.84%) were competitive, 27 (22.6%) were athletes with scheduled activity and 16 (13.4%) were occasional players. The dominant limb was the most affected, with 76 complaints (66.3%), with three bilateral cases.

Reiss and Reiss^15^ reported that most individuals have a lateral dominance and that this in itself can lead to changes between the dominant and non-dominant limbs. A study by Ruwe et al.,^16^ which assessed swimming competitors with shoulder pain, demonstrated a decrease in the upper trapezius activity during sports practice. Another study has found that the latency time of the middle and lower trapezius was higher in patients with impact symptoms than in healthy individuals, suggesting the dominance of the right upper trapezius in these individuals. This imbalance of the scapulothoracic muscle will lead to incoordination of scapular rotation over the thorax.^17^

The current study found statistically significant difference in the pain group at zero degree (p = 0.03), i.e., with the shoulder at rest. In the study by Pimentel et al.,^12^ aimed to evaluate the presence of ligamentous or muscle injury, student-athletes were asked whether they felt comfortable with the arm in resting position (alongside the thorax). Eleven subjects (64.7%) said yes; four (27.5%) reported to feel very comfortable; one (5.8%) said that the arm in resting position produced reasonable comfort; and one (5.8%) reported feeling uncomfortable. These results characterize that 65% of the sample had symptoms of pain in the joint at rest, which can lead to consider the possibility of postural changes in both the current study and in that of Pimentel et al.^12^.

Shoulder pain is one of the most common complaints and disabling of the musculoskeletal system in the general population. It has an estimated prevalence of 15 - 25% in patients that seek orthopedic and physiotherapy facilities.^12^ The literature reports that scapular mobility changes occur in 68% to 100% of individuals with shoulder injury.^7^

The current work shows that the group of patients with pain reported higher pain at angles above 90°, and the 120° angle is the one with higher pain complains, totaling 60%; four reported pain at 90°, totaling 26.67% and two patients reported greater pain at 180° totaling 13.33%. According to Lima and Ollay,^18^ when abduction reaches 90°, it starts generating partial ischemia in myotendinous tissue and, when associated with prolonged isometric periods it may increase the pressure on the supraspinatus muscle, causing increased circulation disorder threshold.

It is known that the complex movement occurs in the three movement planes, however the present study only analyzed scapular positioning in the frontal plane and, to do so, we used highly reliable and reproducible software for angle calculation.^19^ ^-^ ^21^

## CONCLUSION

This is an original study with regard to the approach of scapular dyskinesia in sedentary young adults and in relating it to the presence of shoulder pain. The initial postulate that both would have a significant association was confirmed, because this work has confirmed the influence of positioning alteration of the scapula when associated with shoulder pain in sedentary young adults. Dyskinesia, however, was found in only two of the six angles analyzed (30° and 120°) and only in males, revealing the need for further studies.
